# Biomimetic *versus* enzymatic high-potential electrocatalytic reduction of hydrogen peroxide on a functionalized carbon nanotube electrode†
†Electronic supplementary information (ESI) available. See DOI: 10.1039/c5sc01473e



**DOI:** 10.1039/c5sc01473e

**Published:** 2015-05-22

**Authors:** Bertrand Reuillard, Solène Gentil, Marie Carrière, Alan Le Goff, Serge Cosnier

**Affiliations:** a Univ. Grenoble Alpes , DCM UMR 5250 , F-38000 Grenoble , France and CNRS , DCM UMR 5250 , F-38000 Grenoble , France . Email: alan.le-goff@ujf-grenoble.fr

## Abstract

We report the non-covalent functionalization of a multi-walled carbon nanotube (MWCNT) electrode with a biomimetic model of the horseradish peroxidase (HRP) active site. By modifying the MWCNT electrode surface with imidazole-modified polypyrrole, a new biomimetic complex of HRP was synthesized on the MWCNT sidewalls *via* the coordination of imidazole (Im) to the metal centre of iron protoporphyrin IX, affording **(Im)(PP)Fe^III^**. Compared to the pi-stacking of non-coordinated **(PP)Fe^III^** on a MWCNT electrode, the **(Im)(PP)Fe^III^**-modified MWCNT electrode exhibits higher electrocatalytic activity with an *I*_max_ = 0.52 mA cm^–2^ for the reduction of H_2_O_2_, accompanied by a high onset potential of 0.43 V *vs.* Ag/AgCl. The performances of these novel surface-confined HRP mimics were compared to those of a MWCNT electrode modified by HRP. Although the enzyme electrode displays a higher electrocatalytic activity towards H_2_O_2_ reduction, the **(Im)(PP)Fe^III^**-modified MWCNT electrode exhibits a markedly higher operational stability, retaining 63% of its initial activity after one month.

## Abstract 2

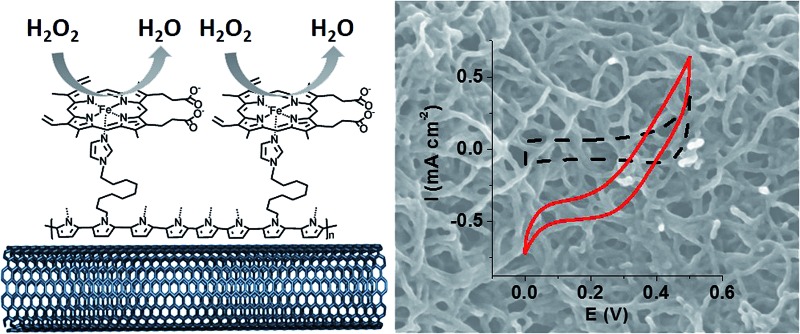
We report the non-covalent functionalization of a multi-walled carbon nanotube (MWCNT) electrode with a biomimetic model of the horseradish peroxidase (HRP) active site.

## Introduction

Horseradish peroxidase (HRP) is the classical enzyme usually used to detect and reduce H_2_O_2_ as it is its natural substrate to detoxify media through oxidation of different natural organic substrates.^1^ The crystal structure of this protein, along with studies of the mechanism of the catalytic cycle, have shown that the catalytic activity is mainly due to the stabilization of an iron oxo bond (Fe^IV^ =O)^2,3^ within its cofactor, iron protoporphyrin IX or hemin. This stabilization is due to the presence of specific amino acids surrounding the active site, two histidine groups (His170 and His42) and an arginine residue (Arg38) stabilize the high oxidation state of the iron metal centre and also act as proton relays. The instability of this high oxidation state compound makes it difficult to study. Nevertheless, the redox potential of this reactive species has been evaluated to be around 0.7 V *vs.* SCE at pH 7 with minor variations according to the protein type.^4^ HRP has been among the most studied enzymes for H_2_O_2_ biosensing applications. Recently, we and others have been able to achieve efficient electron transfer between an electrode and an enzyme for electrocatalytic or biosensing means, leading to high electrocatalytic current densities accompanied by low overpotentials.^5–7^ The reduction of H_2_O_2_ at redox potentials as high as 0.5 V at pH 7, has led to the design of oxygen-reducing biocathodes through the combination of glucose oxidase (producing H_2_O_2_ while oxidizing glucose) and HRP, which performs the reduction of H_2_O_2_ to H_2_O.^8–11^ These oxygen-reducing biocathodes achieve the global 4H^+^/4e^–^ reduction of oxygen with onset potentials of 0.42 V *vs.* SCE at pH 7, which make this bienzymatic system a good candidate to replace high potential multicopper oxidase^12^ in biofuel cell biocathodes.

Interesting approaches have also focused on using inorganic complexes mimicking the HRP active site. For more than 30 years now, porphyrin or phthalocyanin iron(iii) have been used to electrochemically reduce O_2_ and H_2_O_2_ on different types of electrodes.^13–17^ The group of Fukuzumi has recently published several functional H_2_O_2_ fuel cells based on iron porphyrin peroxidase mimics.^18,19^ The possibility of oxidizing H_2_O_2_ at low potential and reducing it at high potential gives the possibility of generating a sufficient voltage, using only H_2_O_2_ as the fuel for both the anode and cathode. However, all these iron porphyrin complexes perform H_2_O_2_ electrocatalytic reduction at lower turnover frequencies and lower potential compared to HRP. This enzyme has been proven to reduce H_2_O_2_ at an overvoltage of 0.55 V compared to the thermodynamic H_2_O_2_/H_2_O redox potential.^5,10^ The difference in catalytic activity is most likely due to the lack of assistance from the surrounding ligands in these complexes compared to the heme enzyme active site. For years now, several groups have also tried to mimic more closely the active site pocket of hemoproteins by modifying the iron porphyrin skeleton or adding an external ligand.^20–23^ For instance, Collman's group has described elegant examples of the reconstitution of the active site of Cytochrome c Oxidase (CcO), where the iron porphyrin is usually directly modified by an imidazole ligand that also helps the complexation of an additional copper centre, forming a dinuclear biomimetic Fe–Cu complex. Similar studies have also focused on synthesized HRP models, either through the direct modification of the hemin core with imidazole residues or by adding another chelating reagent, to bind to the iron cation.^24,25^ All of these contributions clearly show that the nature of the axial ligand coordinating to the iron center is crucial in the stabilization of the high oxidation state species. Axial imidazole ligands mimic the histidine amino acid of the protein in the stabilization of the iron peroxo, hydroperoxo and oxo species.^26–29^

Carbon nanotubes (CNTs) have been widely used for biomimetic catalyst immobilization^30–34^ and especially for electrocatalytic applications. Their ability to immobilize large amounts of redox catalysts and improve heterogeneous electron transfer rates make them a suitable platform to elaborate efficient molecular electrocatalytic materials. CNTs can be functionalized by many different covalent and non-covalent methods increasing the scope of their use, especially for the activation of small molecules. In particular, metal porphyrins display strong pi-stacking interactions with CNTs, which allow their stable and intimate binding^33^ and hence their use in the oxygen reduction reaction.^35,36^ Furthermore, recent studies have reported the covalent functionalization of CNTs by chelating units like pyridine and imidazole groups, enabling the coordination of iron(iii) porphyrin derivatives.^37,38^ These heme enzyme biomimetic models exhibited excellent catalytic properties toward the oxygen reduction reaction, but at high overvoltages of 0.8 V for H_2_O_2_ reduction.^37^

This work describes the synthesis of an original pyrrole-imidazole monomer that enables non-covalent modification of multi-walled carbon nanotubes (MWCNTs) *via* oxidative electropolymerization of the pyrrole subunit. Once the electrode is fully covered by the poly[imidazole-pyrrole] (p-Im) film, the addition of iron protoporphyrin IX, **(PP)Fe^III^**, enables the formation of a biomimetic species through the coordination of the imidazole ligand to the iron(iii) centre. The “on-CNT” synthesis of (imidazole)(protoporphyrinato) iron(iii) (**(Im)(PP)Fe^III^**) HRP biomimetic complex, forming a poly-[(imidazole)(protoporphyrinato) iron(iii)] polymer **p-[(Im)(PP)Fe^III^]**, was then compared with the pi-stacking of free **(PP)Fe^III^** on pristine MWCNT sidewalls and with the direct wiring of HRP immobilized on a MWCNT electrode. We then investigated the performance of these different electrodes towards the electrocatalytic reduction of hydrogen peroxide.

## Results and discussion

### Functionalization of MWCNT electrodes with free (PP)Fe^III^ and p-[(Im)(PP)Fe^III^]

**Fig. 1:**
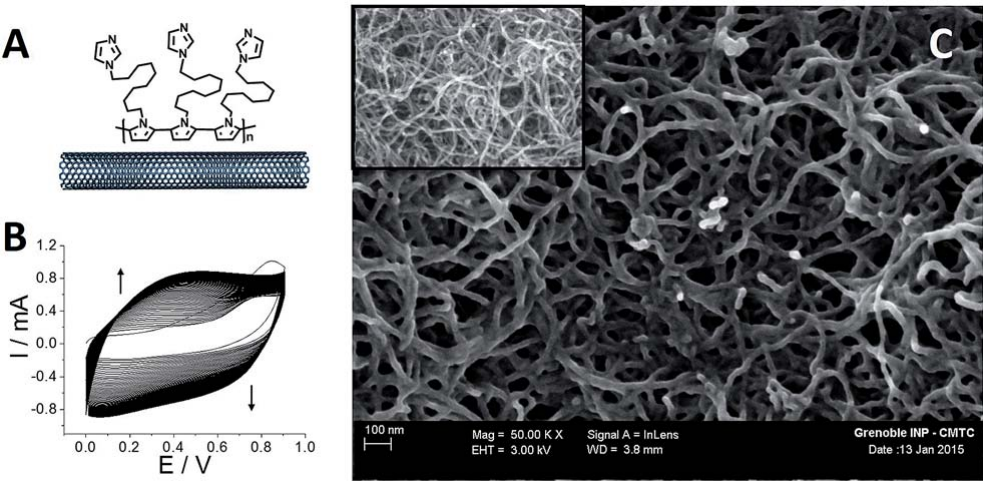
(A) Schematic representation of [poly-imidazole]-pyrrole; (B) oxidative electropolymerization of 1-(11-(1*H*-pyrrol-1-yl)undecyl)-1*H*-imidazole (2 mM) by repeated potential scanning (80 scans) over the range 0–0.9 V in MeCN + 0.1 M TBAP (*v* = 100 mV s^–1^); (C) SEM image of a MWCNT electrode after electrodeposition of [poly-imidazole]-pyrrole, (inset) SEM image of a pristine MWCNT electrode.

The functionalized MWCNT electrodes were prepared in several steps. For the preparation of the poly[imidazole-pyrrole]-functionalized MWCNT electrode (Fig. 1A), p-Im-MWCNT, the modified electrode was first functionalized with the imidazole-pyrrole monomer *via* oxidative electropolymerization performed in acetonitrile (MeCN) + 0.1 M TBAP. Fig. 1B shows the electropolymerization process. 80 CV scans were performed from 0 to 0.9 V *vs.* Ag/AgNO_3_ to ensure full coverage of MWCNT sidewalls with the poly-[imidazole-pyrrole] polymer.

The evolution of the thickness of the poly-[imidazole-pyrrole] film is correlated to the increase of the redox signal at around 0.4 V, corresponding to the electroactivity of the polypyrrole backbone. Fig. 1C displays SEM images of the pristine MWCNT film and the resulting p-Im-MWCNT electrodes. These images confirm the homogenous deposition of a few-nanometer-thin layer of poly[imidazole-pyrrole] on the 10 nm-diameter MWCNTs. It is noteworthy that the highly porous MWCNT nanostructure is preserved during the electropolymerization process.

**Fig. 2:**
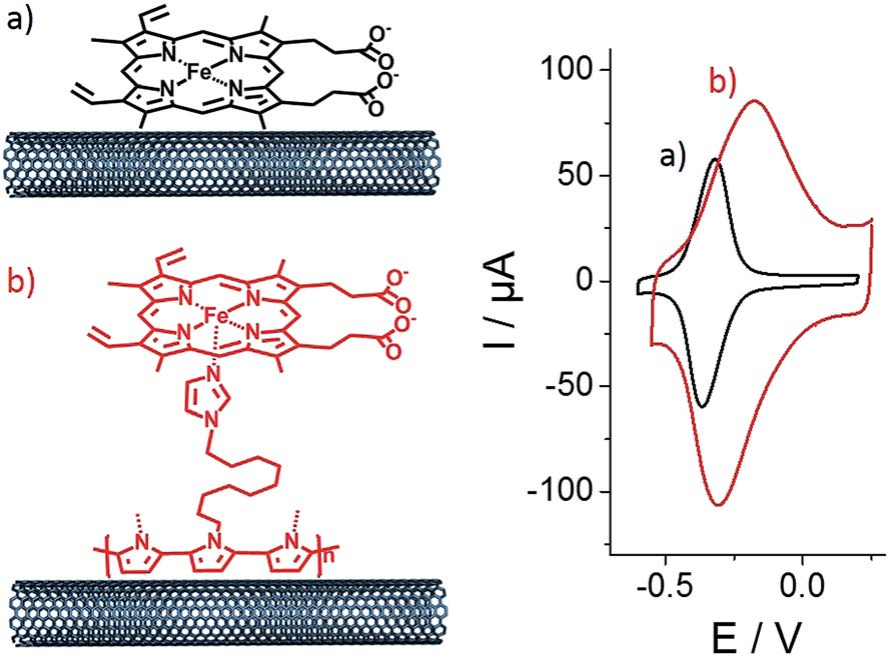
Schematic representation and associated cyclic voltammetry for (a) **(PP)Fe^III^**-functionalized MWCNT electrodes and (b) **p-[(Im)(PP)Fe^III^]**-functionalized MWCNT electrodes in phosphate buffer at pH 7. Electrodes were functionalized by incubation in DMF containing **(PP)Fe^III^** (10 mM), *v* = 10 mV s^–1^.

**Fig. 3:**
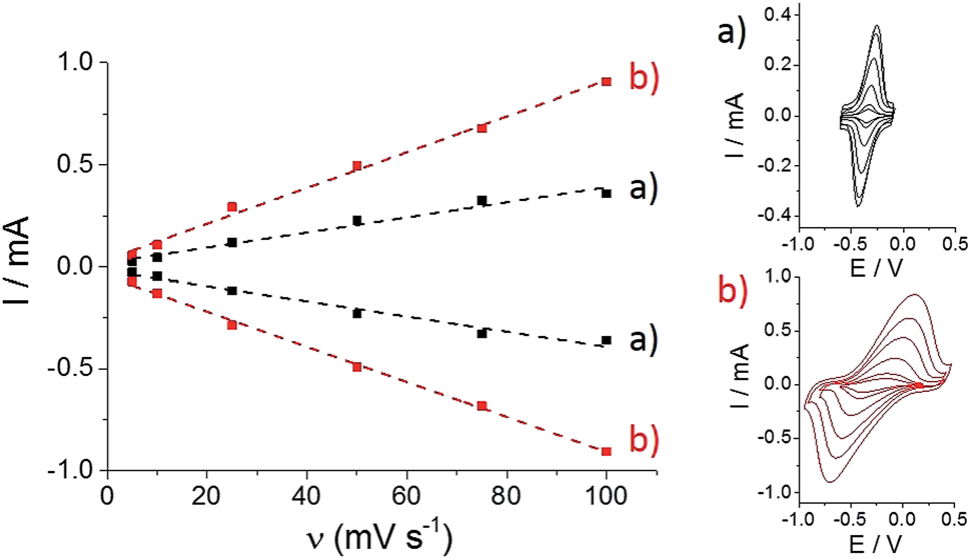
Evolution of the increase in anodic and cathodic peak currents with scan rate for (a) **(PP)Fe^III^**-functionalized MWCNT electrodes and (b) **p-[(Im)(PP)Fe^III^]**-functionalized MWCNT electrodes in phosphate buffer (pH 7).

Then, both pristine and p-Im-MWCNT electrodes were incubated in a 10 mM **(PP)Fe^III^** DMF solution. Cyclic voltammetry was performed in 0.1 M phosphate buffer to investigate the redox response corresponding to the Fe(iii)/Fe(ii) redox couple (Fig. 2). In both cases, one reversible peak system was obtained with *E*_1/2_ = –0.34 V for **(PP)Fe^III^** and –0.24 V for **p-[(Im)(PP)Fe^III^]**. The higher capacitive current for **p-[(Im)(PP)Fe^III^]** arises from the presence of the conjugated polypyrrole film. In addition, these redox systems have a linear dependence of both anodic and cathodic current intensity on scan rate, confirming the immobilization of **(PP)Fe^III^** on the electrodes (Fig. 3).

As observed by CV in Fig. 3, Δ*E* for the Fe(iii)/Fe(ii) redox couple also increases with scan rate. According to the Laviron equation for interfacial electron transfer in adsorbed redox systems,^39^ Δ*E* variations correspond to a *k*_s_ of 0.35 (±0.5) for **(PP)Fe^III^** directly adsorbed on MWCNT sidewalls and 0.12 (±0.5) s^–1^ for **p-[(Im)(PP)Fe^III^]**. The latter exhibits a slower electron transfer rate, which likely arises from the presence of the partially-insulating poly-[imidazole-pyrrole] layer. While **(PP)Fe^III^** is immobilized by taking advantage of the pi–pi interactions between the porphyrin ring and MWCNT sidewalls, coordination of imidazole to the iron centre also allows immobilization of the **(PP)Fe^III^** through the formation of the **[(Im)(PP)Fe^III^]** complex on functionalized MWCNTs. A 100 mV shift towards a positive potential for the **p-[(Im)(PP)Fe^III^]** redox potential is in good agreement with the bottom-up synthesis of an (imidazole)(protoporphyrinato) iron(iii) complex on the electrode.

**Fig. 4:**
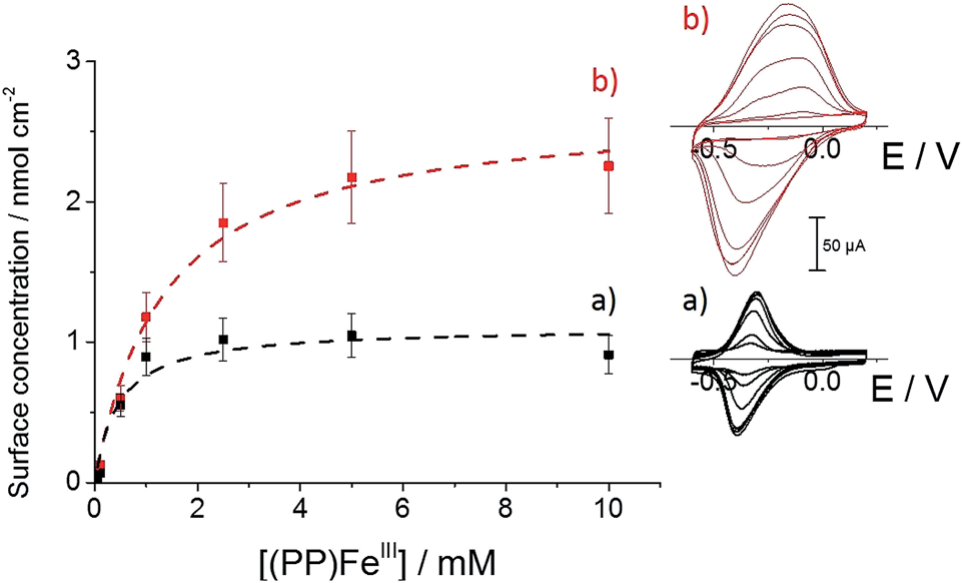
Variation of (a) **(PP)Fe^III^** and (b) **(Im)(PP)Fe^III^** surface coverage with **(PP)Fe^III^** incubation solution concentration in DMF, accompanied by fitting binding isotherms (dashed lines); (inset) associated cyclic voltammetry for (a) **(PP)Fe^III^**-functionalized MWCNT electrodes and (b) **p-[(Im)(PP)Fe^III^]**-functionalized MWCNT electrodes obtained after incubation in a DMF solution of **(PP)Fe^III^** (0.05, 0.1, 0.5, 1, 2.5, 5 and 10 mM) (*v* = 10 mV s^–1^, 0.1 M phosphate buffer, pH 7).

We further investigated the association behavior of **(PP)Fe^III^** by pi–pi interactions and by imidazole ligand coordination on MWCNT surfaces. Fig. 4 shows the influence of the concentration of the **(PP)Fe^III^** incubation solution on the final porphyrin iron(iii) apparent surface coverage. The surface concentrations were estimated by integrating the charge under the anodic or cathodic peak for both of the functionalized-MWCNT electrodes (inset, Fig. 4). For both MWCNT electrodes, the apparent surface coverage of the iron(iii) complex increases with the increasing **(PP)Fe^III^** concentration of the incubation solution. In the case of **p-(Im)(PP)Fe^III^**, a shoulder corresponding to a small redox system around –0.35 V indicates that the pi-stacking of a small amount of **(PP)Fe^III^** on MWCNTs cannot be avoided, despite the presence of the polymer layer.

For both types of electrode, this increase follows a simple Langmuir binding isotherm, according to the equation:
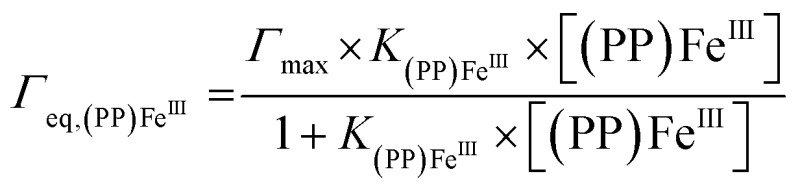
where *Γ*_eq_,**_(PP)Fe^III^_** is the equilibrium surface coverage, *Γ*_max_ is the porphyrin iron(iii) saturating surface coverage and *K* **_(PP)Fe^III^_** is the association constant between **(PP)Fe^III^** and the electrode surface. For the pi-stacking of **(PP)Fe^III^** on pristine MWCNTs, the best fit was achieved with a *Γ*_max_ = 1.1 (±0.2) nmol cm^–2^ and *K* **_(PP)Fe^III^_** = 2220 (±880) L mol^–1^ at 25 °C in DMF. This association constant is similar to that previously reported (1900 L mol^–1^) at 25 °C in DMF for a pyrene-modified ruthenium complex immobilized by pi-stacking on similar MWCNT electrodes.^32^ The formation of **p-[(Im)(PP)Fe^III^]** leads to the following values: *Γ*_max_ = 2.7 (±0.4) nmol cm^–2^ and *K* **_(PP)Fe^III^_** = 749 L mol^–1^. The association constant of 750 (±120) L mol^–1^ for the binding of **(PP)Fe^III^** to surface-confined imidazole ligands can be related to the association constant of 128 L mol^–1^ measured for the binding of *N*-methylimidazole to a tetraphenylporphyrin iron(iii) complex measured in DMF solution.^40^ The higher maximum surface coverage *Γ*_max_ for **p-[(Im)(PP)Fe^III^]**, compared to pi-stacked **(PP)Fe^III^**, likely arises from the fact that the poly-[imidazole-pyrrole] provides a more accessible binding site for **(PP)Fe^III^** binding compared to pristine MWCNT sidewalls for the pi-stacking of free **(PP)Fe^III^**. It is noteworthy that formation of **p-[(Im)(PP)Fe^III^]** on the electrode surface requires several hours to reach the equilibrium surface coverage while pi–pi stacking of **(PP)Fe^III^** only requires a few minutes to reach equilibrium.

### Electrocatalytic H_2_O_2_ reduction: biomimetic *vs.* enzymatic electrocatalysis

**Fig. 5:**
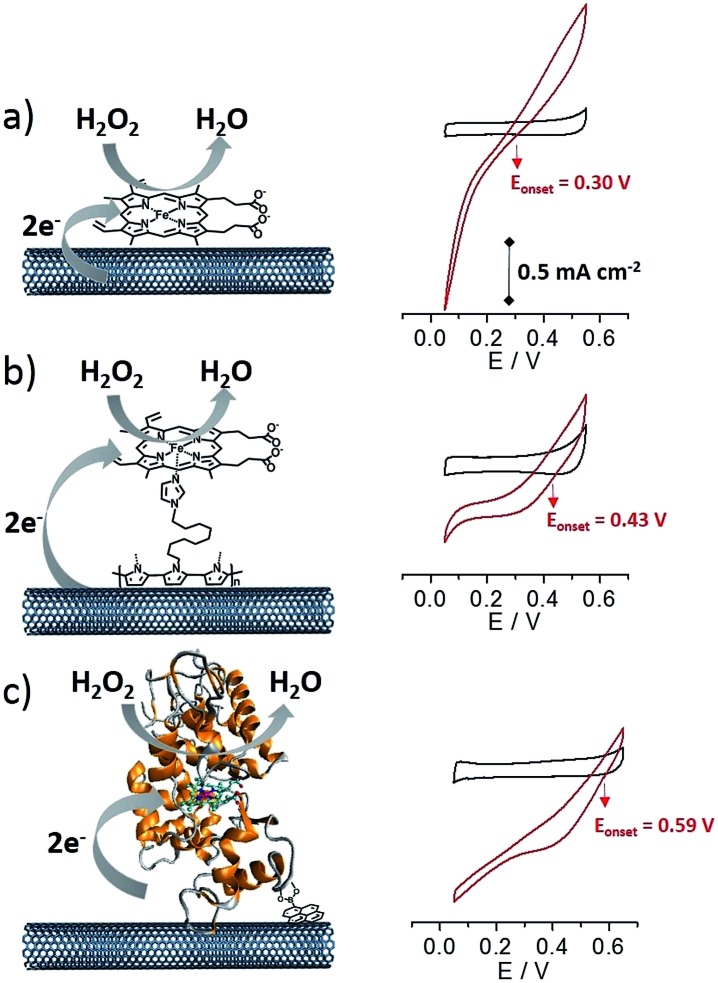
Cyclic voltammetry for (a) **(PP)Fe^III^**-functionalized MWCNT electrodes, (b) **p-[(Im)(PP)Fe^III^]**-functionalized MWCNT electrodes and (c) HRP-functionalized MWCNT electrodes in the absence and presence of 10 mM H_2_O_2_ (*v* = 10 mV s^–1^, phosphate buffer, pH 7).

The electrocatalytic properties of these modified MWCNT electrodes were investigated towards H_2_O_2_ reduction. The performances of these biomimetic MWCNT electrodes were compared with those of MWCNT electrodes functionalized with HRP. This enzyme was immobilized through the formation of a boronic ester covalent linkage with a pyrene-boronic-acid derivative.^10^ Fig. 5 displays the CV data for the three different MWCNT electrodes in the presence of 10 mM H_2_O_2_.

**Fig. 6:**
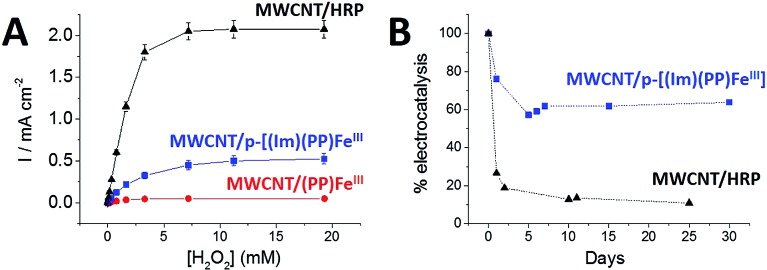
(A) Cathodic catalytic current densities as a function of H_2_O_2_ concentration recorded at the () **(PP)Fe^III^**-functionalized MWCNT electrode, (■) **p-[(Im)(PP)Fe^III^]**-functionalized MWCNT electrode and (▲) HRP-functionalized MWCNT electrode. Chronoamperometric experiments were performed at a fixed potential of 0.3 V in 0.1 M phosphate buffer pH 7. (B) Evolution of the maximum catalytic current density for the (■) **p-[(Im)(PP)Fe^III^]**-functionalized MWCNT electrode and the (▲) HRP-functionalized MWCNT electrode as a function of time. Error bars were estimated from experiments performed on three electrodes.

All three electrodes exhibited electrocatalysis of both H_2_O_2_ reduction and oxidation. Onset potentials of +0.30 (±0.01), +0.43 (±0.02) and +0.59 (±0.02) V were measured for **(PP)Fe^III^**, **p-[(Im)(PP)Fe^III^]** and HRP respectively (see Fig. 4 and Table 1). Chronoamperometric measurements were performed at a fixed potential of 0.30 V in the presence of different concentrations of H_2_O_2_ (Fig. 6A). As expected, the HRP-based electrode was able to reduce H_2_O_2_ at low overpotentials with an excellent maximum current density of 2.1 mA cm^–2^. The **p-[(Im)(PP)Fe^III^]**-based electrode exhibited an onset potential of 0.43 (±0.02) V, representing an intermediate value between the **(PP)Fe^III^**-based electrode and the HRP-based electrode and confirming that coordination of imidazole results in the increase in the redox potential of the Fe(iv)=O intermediate.

**Table 1:** Electrochemical parameters of the **(PP)Fe^III^**-, **p-[(Im)(PP)Fe^III^]**- and HRP-functionalized MWCNT electrodes

MWCNT electrode	*E*_1/2_(Fe^III^/Fe^II^) (V)	*E*_onset_ (V)	*I*_max_ at 0.3 V (mA cm^–2^)
**(PP)Fe^III^**	–0.34 (±0.01)	+0.30 (±0.01)	0.050 (±0.003)
**p-[(Im)(PP)Fe^III^]**	–0.24 (±0.01)	+0.43 (±0.02)	0.52 (±0.06)
HRP	–0.10	+0.59 (±0.02)	2.1 (±0.1)

Furthermore, **p-[(Im)(PP)Fe^III^]** exhibited an excellent current density for H_2_O_2_ reduction of 0.52 mA cm^–2^ accompanied by excellent stability (Fig. 6B). An apparent turnover frequency (TOF) of 4.8 s^–1^ was estimated from the **(Im)(PP)Fe^III^** surface coverage. Although the **p-[(Im)(PP)Fe^III^]**-based electrode exhibits a lower maximum current density (0.52 mA cm^–2^) for H_2_O_2_ reduction than the HRP-based electrode, the biomimetic electrode displays a markedly better operational stability. It appears that the HRP-based electrode retains only 11% of its initial electroactivity after 25 days whereas the **p-[(Im)(PP)Fe^III^]**-functionalized MWCNT electrode still retains 63% of its initial activity after one month (Fig. 6B).

## Conclusions

These functionalized MWCNT electrodes, from the pi-stacked free **(PP)Fe^III^** cofactor to the immobilized directly-wired HRP, all have in common the surface-confined **(PP)Fe^III^** core. The functionalization of MWCNTs with an imidazole-modified polymer allows both the immobilization of **(PP)Fe^III^** and the modification of the iron(iii) coordination sphere. The electrochemistry of these functionalized MWCNTs gets closer to the electrochemistry of HRP-based electrodes in terms of the redox potential of the Fe(iii)/Fe(ii) couple and the redox potentials of the Fe(iv)=O intermediate, as confirmed by the onset potentials of the cathodic electrocatalytic wave in the presence of H_2_O_2_. Thanks to the high conductivity and high electroactive surfaces, these biomimetic electrodes constitute an efficient tool for the electrocatalytic reduction of H_2_O_2_. Furthermore, the improved stability of the biomimetic catalyst over weeks represents an important advantage in the design of a new generation of biocathodes and hence biofuel cells. However, there is still room for improvement to approach the true redox potential of the HRP Fe(iv)=O catalytic intermediate. This could especially be achieved by providing a proton source in the vicinity of the iron centre. Thanks to the flexibility of these functionalization techniques, this work is underway, especially in the design of more sophisticated pyrrole monomers and porphyrin ligands.
