# Supplementary material for: Biomimetic versus enzymatic high-potential electrocatalytic reduction of hydrogen peroxide on a functionalized carbon nanotube electrode
Source: Chem Sci. 2015 May 22;6(9):5139–43. doi: 10.1039/c5sc01473e (PMC5666682; doi:10.1039/c5sc01473e)
Supplement: Supplementary file 1 [file SC-006-C5SC01473E-s001.pdf]

Electronic Supplementary Material (ESI) for Chemical Science.
This journal is © The Royal Society of Chemistry 2015
Please do not adjust margins

Journal Name

SUPPORTING INFORMATIONS

Experimental

Materials

Acetonitrile (MeCN) (HPLC grade) used for electrochemistry was obtained from VWR chemicals and used without further purification. Tetrabutylammonium perchlorate (Fluka) $[Bu_4N]ClO_4$ (TBAP) was used as supporting electrolyte in organic media. All reagents, chemicals products and enzyme, purchased from Aldrich, were of reagent grade quality and used as received unless it is mentioned. NMR spectra were recorded on a Bruker AVANCE 400 operating at 400.0 MHz for ^1H. ESI mass spectra were recorded with a Bruker APEX-Qe ESI FT-ICR mass spectrometer.

Electrochemistry Measurements

The electrochemical experiments in organic media were performed in MeCN in a three-electrode electrochemical cell under a dry argon atmosphere and in a glovebox ($[O_2]$ < 20 ppm) using in both cases an Autolab pgstat100 potentiostat. The surface of GC electrodes were polished with a 2 µm diamond paste purchased from Presi (France) and rinsed successively with water, acetone, and ethanol. A Pt wire placed in a separated compartment was used as counter electrode, and the Ag/$AgNO_3$ 10 mM in MeCN + TBAP (0.1 M) served as a reference electrode in organic media.
For electrochemical experiments performed in aqueous media, a Biologic VMP3Multi Potentiostat was used. Experiments were performed in 0.1 M phosphate buffer (pH 7). A platinum grid was used as the counter electrode and an SCE served as the reference electrode.
Potentials in organic and aqueous media are referred to the (Ag/$AgNO_3$) electrode and the (Ag/AgCl) electrode, respectively.
Data fitting was performed with OriginPro9.
The morphology of the modified MWCNTs electrodes was investigated by SEM using an ULTRA 55 FESEM based on the GEMENI FESEM column with beam booster (Nanotechnology Systems Division, Carl Zeiss NTS GmbH, Germany) and tungsten gun.

Synthesis

1-(11-bromoundecyl)-1H-pyrrole was synthesized according to previously described procedures[1].
Synthesis of 1-(11-(1H-pyrrol-1-yl)undecyl)-1H-imidazole (imidazole-pyrrole): 340 mg of imidazole (5 mmol) and 280 mg of K_2CO_3 (2 mmol) were dissolved in 30 mL of MeCN. Then, 150 mg of 1-(11-bromoundecyl)-1H-pyrrole (0.5 mmol) were added to the mixture. The reaction mixture was then refluxed under argon atmosphere for 24h. The suspension was then filtered and the solvent removed under vacuum. The crude product was then extracted with 60 mL of CH_2Cl_2 and washed with H_2O saturated with K_2CO_3 (3x50 mL). The organic phase was then dried over $MgSO_4$ and under vacuum to yield to 130 mg of a pale yellow oil as the pure product (90% yield).
^1H NMR: δH/ppm (400 MHz, $CDCl_3$): 1.20-1.30 (m, 14H), 1.75 (q, j = 6.4Hz,2H), 1.82 (q, J = 7.2Hz, 2H), 3.85 (t, J = 7.2 Hz, 2H), 4.11 (t, J = 7.6 Hz, 2H), 6.12 (t, J = 2Hz, 2H), 6.64 (t, J = 2Hz, 2H), 7.02 (s, 1H), 7.25 (s, 1H), 8.46 (s, 1H).
^{13}C NMR: δC/ppm (400 MHz, $CDCl_3$): 26.33, 26.73, 28.92, 29.13, 29.27, 29.32, 29.33, 29.36, 30.6, 31.52, 48.59, 49.6, 107.74 and 120.43.
MS (ESI+): 288^+ $(M+H^+)$

Preparation of MWCNT electrodes

Commercial grade thin multi-walled carbon nanotubes (9.5 nm diameter, purity >95%) were obtained from Nanocyl and used as received without any purification step. MWCNT films were prepared by drop coating 20 µL of a 5 mg mL^{-1} dispersion of MWCNTs in N-methyl pyrrolidinone (NMP) onto the glassy carbon (GC) electrode surface. The deposit was then allowed to dry under vacuum.

Functionalization of Electrodes with polypyrrole-imidazole (p-Im) and p-[(Im)(PP)Fe^{III}]

The p-Im-modified MWCNTs electrode (p-Im-MWCNTs) was obtained by electrodeposition of the imidazole-pyrrole monomer from a 2 mM solution in MeCN 0.1 M TBAP. In order to saturate the surface with the p-Im polymer, 80 scans between 0 and 0.9 V Vs Ag/$AgNO_3$ were performed.
The pi-stacking of **(PP)Fe^{III}** onto the MWCNT-modified electrode was performed by incubating the non-functionnalized electrode in a solution of **(PP)Fe^{III}** in DMF (0.05 ; 0,1 ; 0,5 ; 1 ; 2.5 ; 5 and 10 mM) for several minutes. In order to generate the formation of the p-[**(Im)(PP)Fe^{III}**], the same procedure was repeated with p-Im-MWCNT-modified electrode with an incubating time of 3 hours.

References

1 J. S. Lee, Y. S. Chi, J. Kim, W. S. Yun and I. S. Choi, *Phys. Chem. Chem. Phys.*, 2008, **10**, 3138–3149.

Please do not adjust margins
